# Chitosan Derivatives: Challenges and Opportunities in the Green and Sustainable Transition Era

**DOI:** 10.3390/molecules31081273

**Published:** 2026-04-13

**Authors:** Ana Morais, Rita Lima, Madalena M. M. Pinto, Maria Elizabeth Tiritan, Carla Fernandes

**Affiliations:** 1 Laboratório de Química Orgânica e Farmacêutica, Departamento de Ciências Químicas, Faculdade de Farmácia, Universidade do Porto, Rua Jorge Viterbo Ferreira 228, 4050-313 Porto, Portugal; moraisana2001@hotmail.com (A.M.); ritaalexandralima@gmail.com (R.L.); madalenakijjoa@gmail.com (M.M.M.P.); beth@ff.up.pt (M.E.T.); 2 CIIMAR/CIMAR LA—Interdisciplinary Centre of Marine and Environmental Research, University of Porto, Terminal de Cruzeiros do Porto de Leixões, Avenida General Norton de Matos, s/n, 4450-208 Matosinhos, Portugal; 3 1H-TOXRUN—One Health Toxicology Research Unit, University Institute of Health Sciences (IUCS), CESPU—CRL, 4585-116 Gandra, Portugal

**Keywords:** chitin, chitosan, chitosan derivatives, eco-friendly, green chemistry, sustainable chemistry, click chemistry

## Abstract

Transition towards sustainable and environmentally friendly practices within the field of chemistry and materials science has become essential in light of current environmental challenges. This review provides a comprehensive overview of the challenges and opportunities in the various steps involved in producing chitosan derivatives, with particular emphasis on eco-friendly strategies. Key methodologies for chitin isolation from diverse natural sources, chitin deacetylation, and the chemical modification of chitosan are discussed, integrating green chemistry principles and eco-efficient processes. Advances in sustainable technologies that prioritize cost-effectiveness, safety, and performance are highlighted. The importance of interdisciplinary collaboration, innovative isolation and purification strategies, the adoption of continuous-flow processes, and greener synthetic approaches, such as click chemistry, are also explored. Overall, this work supports the adoption of a holistic approach for the development of chitosan derivatives, contributing to more sustainable and environmentally responsible materials and production processes.

## 1. Introduction

In recent years, increasing attention has been directed toward the development of environmentally benign methodologies for the synthesis of novel materials, including polymers and biopolymers such as polysaccharides. These materials, owing to their renewable origin and biodegradability, represent promising candidates for sustainable industrial applications [1,2].

Polysaccharides are a class of biomacromolecules with diverse biological activities and applications [3]. Their bioactivities are largely determined by their chemical structure, including monosaccharide composition, inter-residue linkages, and three-dimensional conformation [4]. Many studies have reported that chemical modifications of polysaccharides significantly enhance their structural diversity, thereby amplifying and/or potentially introducing novel bioactivities and applications [5,6].

**Figure 1:**
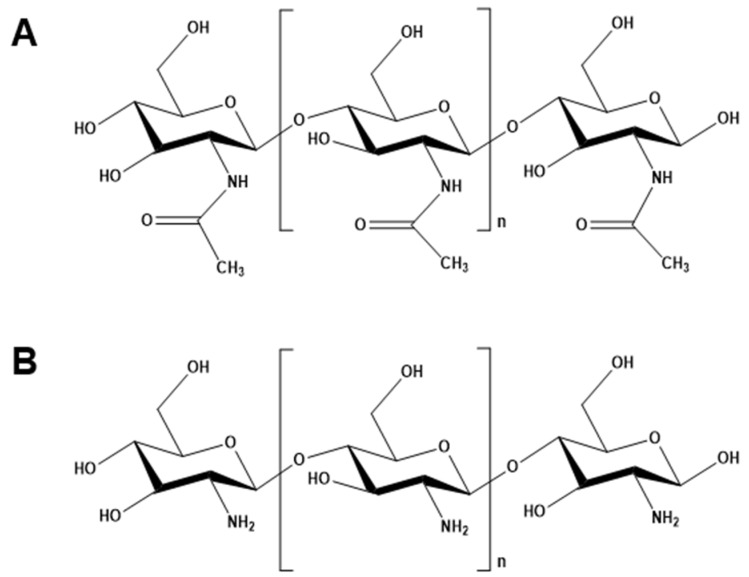
Structure of chitin (**A**) and chitosan (**B**).

Chitin, the second most abundant polysaccharide on earth, is a linear polysaccharide of the amino sugar *N*-acetylglucosamine, featuring an acetyl group attached to the amino group of each glucose unit (Figure 1A) [7]. The acetyl groups impart distinctive physicochemical characteristics to chitin. Owing to its unique structure, chitin exhibits remarkable physical, chemical, mechanical, and optical properties, thereby facilitating the attainment of adjustable and exceptional traits, including low density, high porosity, renewability, natural biodegradability, and environmental compatibility [8]. Furthermore, its surface functionality can be tailor-made for various specific applications [7,8].

The most important derivative of chitin is chitosan (Figure 1B), a linear polysaccharide composed of randomly distributed D-glucosamine and *N*-acetyl-D-glucosamine units [9]. Discovered in 1859 by the physiologist Charles Rouget, chitosan was first obtained by refluxing chitin in potassium hydroxide, yielding a novel product soluble in aqueous acidic media [10]. Indeed, chitin is converted to chitosan through deacetylation. Structurally, chitosan features an amino group at C2 and hydroxyl groups at C3 and C6, which play a crucial role in forming intermolecular hydrogen bonds that determine the polymer’s stability [11]. The composition and structural characteristics of chitosan chains vary depending on their source and the method used for chitin deacetylation [12]. Solid chitosan appears as a semi-crystalline polymer with a white to slightly yellow coloration. Particularly, chitosan is insoluble in aqueous, alkaline, and organic solvents, but is soluble in diluted acidic solutions below a pH threshold of 6.3 [13]. Paramount for the comprehensive characterization of chitosan is the determination of its deacetylation degree (DD), which inherently governs its intrinsic properties and functional applications [6].

In addition to its broad spectrum of biological properties [13], the versatility and environmentally benign nature of chitosan have driven extensive research toward the development of sustainable, efficient, and scalable synthesis and processing methodologies to replace conventional, non-eco-friendly procedures [14]. Furthermore, chitosan derivatization has been widely investigated to enhance solubility, stability, and functional performance, as well as to introduce tailored properties that broaden its application scope [9]. Despite these advances, significant challenges remain, including variability in raw material sources, energy- and chemical-intensive extraction processes, and limited control over physicochemical properties [11].

This review outlines the principal steps in the development of chitosan derivatives, including chitin isolation, chitosan production, and subsequent derivatization strategies. Particular emphasis is placed on environmentally friendly extraction methods for chitin isolation and on green, sustainable deacetylation approaches for chitosan production. Furthermore, the review highlights innovative and eco-friendly modification techniques, including click chemistry, that enable efficient functionalization of chitosan while minimizing environmental impact.

Framed within the broader context of green and sustainable chemistry, this review provides a comprehensive and up-to-date overview of recent advances in the field. By critically analyzing emerging methodologies and environmentally conscious strategies, it offers novel insights into current trends and supports the advancement of more sustainable pathways for the design and application of chitosan-based materials.

To select the relevant papers, research was conducted in the SCOPUS database using keywords related to the scope of the review, namely, chitin or chitosan or chitosan derivatives and isolation or acetylation or derivatization or click-chemistry or green strategies or applications, searched in “Article title, Abstract, Keywords”. The selection of studies took place in January of 2026.

## 2. From Chitin to Chitosan: Challenges and Opportunities

Chitin has attracted considerable interest within the scientific community owing to its abundance and low cost, as it is found in the extracellular matrix of numerous invertebrates [15]. It can be isolated from crustaceans (lobsters, shrimp…), insects (scorpions, ants, cockroaches, spiders, beetles, brachiopods), invertebrate animals, or mollusks (octopus, cuttlefish, clams, oysters, geoducks, fossils, squids, fossils, snails), algae (diatoms, brown algae, green algae), and certain microorganisms (fungi and bacteria) [16]. Chitin is generally a key structural component of protective or supportive extracellular matrices that cover the tissues that produce it or, in some organisms, the entire body [8].

### 2.1. Main Sources of Chitin

#### 2.1.1. Crustaceans

Several studies have emphasized crustaceans as the primary commercial sources of chitin [17]. Due to the challenges posed by the extreme seasonality and limited availability of crustacean shells, coupled with the high cost and inconsistent physical and chemical characteristics, such as protein contamination, variable levels of deacetylation, and high molecular weight, the traditional method of chitin production from crustaceans has limited potential for widespread industrial adoption [18,19].

To address these economic and environmental issues, a viable solution was to convert crustacean shellfish waste into high-value products. Utilizing this waste to produce valuable building blocks for various compounds offers an opportunity to both reduce environmental impact and generate new sources of revenue [20].

The global production of crustaceans for human consumption is estimated at approximately 8 million tons per year, of which about 40% comprises waste exoskeletons. These by-products contain between 15% and 40% chitin, representing a significant potential source of this biopolymer [21]. However, the industrial exploitation of crustaceans typically begins in the spring after the spawning season, resulting in a seasonal supply of fishery waste [17]. Moreover, the long-term sustainability of crustacean cultivation is currently under debate [22,23,24].

#### 2.1.2. Fungi

Fungi represent the second most prevalent source of chitin following crustaceans [25,26]. Chitin is a prevalent component within various fungal groups [27]. It serves as a structural constituent of fungal membranes, cell walls, mycelial structures, stalks, and spores. Chitin is not universally present in all fungal species but is rather confined to certain taxa, including *Basidiomycetes*, *Ascomycetes*, *Zygomycetes*, and *Deuteromycetes* [27]. Within *Saccharomyces cerevisiae*, chitin is notably recognized as a primary element within the septa of both mother and daughter cells [28]. The exploration of certain mushroom species, such as *Amanita phalloides*, *Conocybe filaris*, *Cortinarius species*, *Galerina marginata*, *Amanita species*, and *Lepiota brunneoincarnata*, as viable commercial sources of chitin warrants careful consideration, given their significant toxicity [29]. Chitin derived from fungi exhibited a composition akin to that found in crustaceans [30]. Additionally, chitin derived from fungi cultivated under controlled conditions is more likely to offer reliability, and the product reveals consistent characteristics. Sources of fungal chitin are readily accessible throughout the year, and biomass materials can be harvested inexpensively through simple fermentation processes [25]. Fungal biomass contains lower chitin contents compared to crustacean shells, generally in the range of 10–20% of dry weight, but offers advantages in terms of process sustainability. Controlled fermentation enables consistent production independent of seasonal variability, and milder extraction conditions can reduce environmental impact [31,32].

Given the increasing demand for chitin and chitosan, there is a growing need for the industrial-scale production of fungal species to meet various competing priorities and ensure an adequate supply for chitin manufacturing.

#### 2.1.3. Insects

In addition to crustaceans and fungi, insects are also a promising source of chitin, though they have received relatively little attention in the past [33]. They have recently emerged as a promising alternative source, with chitin yields typically ranging from 5 to 15% depending on species and developmental stage [34,35]. Insects offer several advantages compared to crustaceans, as they are not subject to seasonality and can be readily bred due to their high fertility and reproductive rate. Furthermore, insect rearing facilities are being established worldwide [36].

Insect farming demonstrates improved environmental performance, including reduced greenhouse gas emissions, lower water usage, and high feed conversion efficiency compared to traditional livestock and marine sources. Furthermore, the scalability of insect production is supported by modular and vertical farming systems, as well as the valorization of organic side streams [37].

Presently, insect husbandry is undergoing significant expansion, primarily to sustain animal feed production [38]. This surge in insect cultivation increases access to substantial volumes of insect biomass and chitin-rich industrial by-products, including exuviae and exoskeletons [23]. Consequently, commercial insect processing not only addresses the challenge of ensuring a consistent supply of chitin but may also address malnutrition in human diets.

Life cycle assessment studies indicate that while crustacean-derived chitin benefits from established supply chains, fungal and insect sources offer significant opportunities for more sustainable and circular production systems [23]. Nevertheless, challenges remain in optimizing extraction yields, ensuring batch-to-batch consistency, and developing greener, cost-effective processing technologies.

### 2.2. Isolation of Chitin from Natural Sources

**Figure 2:**
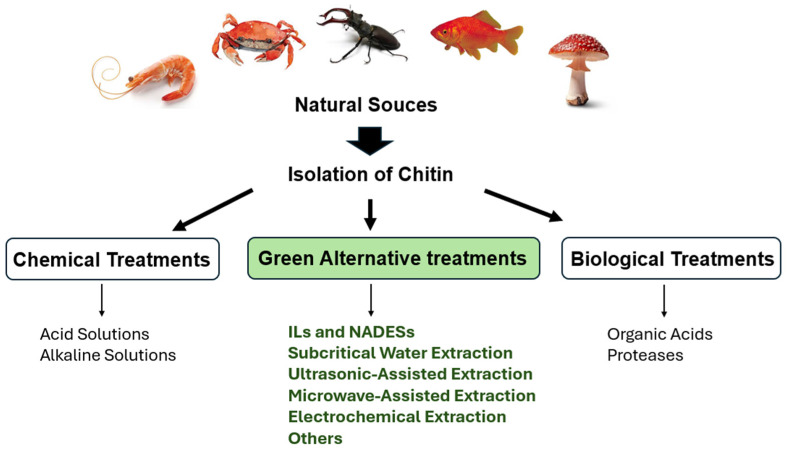
Isolation of chitin through chemical, biological, and green methodologies. ILs: Ionic liquids NADESs: Natural deep eutectic solvents.

Chitin can be extracted through diverse methods, as illustrated in Figure 2. They include chemical methods, typically using strong acids and alkaline solutions, or biological methods, such as fermentation or enzymes [39]. Each approach, whether chemical or biological, has its own advantages and disadvantages [40]. Beyond classic strategies, increasing attention has been directed toward the development and implementation of environmentally friendly and sustainable approaches for chitin extraction and processing [41]. Currently, alternative eco-friendly chemical treatments, such as natural deep eutectic solvents (NADESs), and green methodologies, such as electrochemical extraction, are being investigated for chitin recovery [42,43].

#### 2.2.1. Chemical Treatment

**Figure 3:**
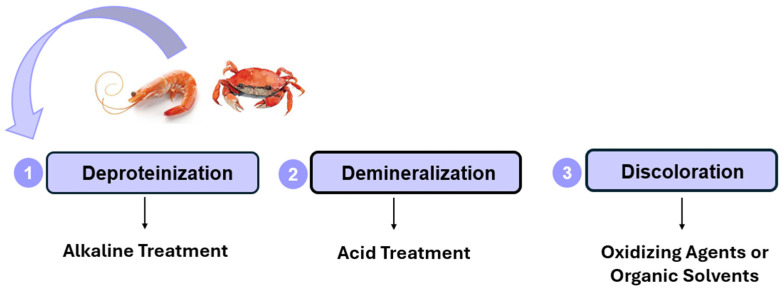
General strategy for isolation of chitin from crustacean shells by chemical methods.

A general strategy for chitin extraction and processing from natural sources, such as crustacean shells, using a chemical approach, is illustrated in Figure 3. It typically comprises three main steps: deproteinization with an alkaline solution, demineralization with an acid solution, and, when required, discoloration to remove residual pigments [44].

The chitin source undergoes washing to remove adhering impurities, drying, and grinding to increase surface area prior to chemical treatment [45]. This includes the use of a strong alkaline solution, such as hydrolysis with NaOH at high temperatures and concentrations, which leads to the breakdown of polymeric chains and results in a high degree of chitosan deacetylation [46,47].

Deproteinization involves breaking the chemical bonds between proteins and chitin, using chemicals to depolymerize the biopolymer [48]. Efficient protein removal is crucial, particularly for biomedical and pharmaceutical applications, both to ensure high purity and to mitigate allergenic responses associated with residual shellfish proteins [49]. In conventional chitin extraction, deproteinization entails the use of strong bases and acids at high temperatures [50,51]. This process demands high energy consumption and generates effluents with high chemical concentrations, requiring appropriate treatment for neutralization [52]. The intensive use of strong reagents also increases process costs and can compromise product purity and performance due to side reactions, oxidation, and excessive depolymerization [53].

Demineralization is primarily intended to remove inorganic components, especially calcium carbonate and, to a lesser extent, calcium phosphate and other mineral salts. This step is typically performed using strong acids such as H_2_SO_4_, HCl, CH_3_COOH, HNO_3_, and HCOOH [54]. Demineralization occurs by the decomposition of calcium carbonate into calcium chloride, releasing carbon dioxide [55]. Process variables such as acid concentration, temperature, solid-to-liquid ratio, and contact time must be carefully controlled to achieve efficient decalcification while minimizing excessive hydrolysis of the polysaccharide chains [56].

In some cases, discoloration is also done to remove residual pigments, such as carotenoids and other colored compounds that remain associated with the chitin matrix [56]. This can involve oxidizing agents or organic solvents, applied under controlled conditions to avoid significant degradation of the polymer backbone. Discoloration markedly improves the appearance and perceived purity of chitin and is especially important when the material is intended for high-value applications in biomedical, pharmaceutical, or food-related fields [19,57,58]. For instance, Younes and Rinaudo [57] reported that light-colored chitin and chitosan exhibit improved biocompatibility and are preferred for biomedical applications, where the absence of colored impurities can affect regulatory compliance and user acceptance. In food applications, Arbia et al. [19] showed that decolorization reduces residual pigments and off-flavors, enhancing sensory quality. Similarly, Zhang et al. [58] demonstrated that controlled decolorization in large-scale chitin production improves material consistency and functional performance in applications, such as antioxidant films and nutraceuticals. Overall, decolorization contributes not only to appearance but also to the functional quality and applicability of chitin and chitosan in high-value sectors.

#### 2.2.2. Biological Treatment

**Figure 4:**
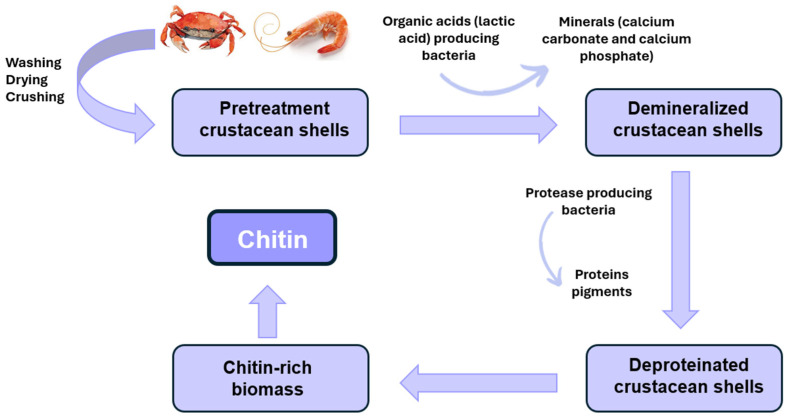
General strategy for isolation of chitin from crustacean shells by biological methods.

Biological extraction involves the use of microorganisms that produce enzymes and organic acids at relatively low cost, thereby promoting a cleaner, more sustainable process and favoring the production of high-quality chitin (Figure 4) [59,60]. The biological process has become more attractive due to the production of high-quality products at affordable costs and without generating high concentrations of chemical effluents, as observed in the chemical process [61]. Commonly employed biological methods for chitin extraction include enzymatic deproteinization and fermentation using microorganisms [62,63].

##### Enzymatic Processes in Biological Extraction

Enzymes, particularly proteases, play a central role in biological chitin extraction. They hydrolyze proteins under mild pH and temperature conditions (Figure 4), preventing the formation of environmentally harmful by-products and preserving the structural integrity of chitin with minimal risk of deacetylation [52,64,65].

Common proteases used for deproteinization include papain, trypsin, pepsin, alcalase, and pancreatin, which differ in hydrolytic efficiency and optimal operating conditions [66]. Alcalase, a bacterial alkaline protease, typically exhibits higher degrees of hydrolysis (DH) under alkaline pH (8.0–9.0) and moderately elevated temperatures (50–55 °C). In contrast, papain, a plant-derived cysteine protease, operates over a broad pH range (5.0–7.0) and moderate temperatures (37 °C), but generally yields lower DH. Pepsin is most active at acidic pH (1.6–2.5) and moderate temperature (37 °C), efficiently hydrolyzing denatured proteins but with limited activity on native structures at neutral pH. Trypsin exhibits optimal activity at basic pH (7.5–8.5) and 37 °C, with substrate specificity for lysine and arginine residues, while pancreatin, a mixture of digestive enzymes, shows broader but less specific proteolytic activity under neutral to mildly alkaline conditions [66].

Proteases produced by lactic acid bacteria, particularly under low-pH fermentation conditions, can also contribute to protein hydrolysis, although their specific activity and optimal conditions tend to vary widely depending on the strain and fermentation environment [67].

The use of proteases enables the recovery of value-added by-products, such as proteins, enzymes, and pigments, which find applications in the food industry [68]. Reaction times generally range from 2 to 8 h, though some enzymatic processes may extend up to 24 h [69].

Bacteria are key sources of industrial proteases due to high productivity, short fermentation cycles, and low production cost. Species of *Bacillus* produce neutral and alkaline proteases with notable thermo- and pH stability, as well as tolerance to organic solvents, detergents, and oxidizing agents. These enzymes typically show optimal activity at 50–70 °C and retain > 80% activity after 1 h at 60 °C. They are active over a broad pH range (6–11), with some alkaline proteases remaining stable even at pH 12. Such properties make *Bacillus* proteases highly suitable for applications in detergents, leather processing, and the food and pharmaceutical industries, where harsh operating conditions are common.

##### Lactic Acid Fermentation-Mediated Chitin Extraction

Recent studies have identified biological methods to extract chitin from crustacean shell waste using organic acid-producing bacteria and specific enzymes. Lactic acid, produced from the breakdown of glucose, creates the acidic conditions needed to preserve shellfish waste through a process known as ensiling. This low pH environment inhibits the growth of spoilage-causing microorganisms in shrimp waste [70]. 

The process of fermenting with lactic acid bacteria has been successfully applied to various shellfish by-products, including shrimp shells, scampi shells, crayfish exoskeletons, and prawn shells [71,72,73]. The lactic acid produced reacts with calcium carbonate, a common component of chitin, forming calcium lactate (Figure 4), which can be removed by washing. This step, called demineralization, results in the extraction of chitin [63].

##### Non-Lactic Acid Fermentation-Mediated Extraction

In an effort to optimize these techniques, researchers have explored alternative bacterial fermentation methods, beyond lactic acid fermentation, for chitin recovery from crustacean shells [63]. These studies have also investigated the use of proteolytic enzymes for the deproteinization of crustacean shells [74].

Recent research has successfully isolated and identified various bacteria that produce proteases. These proteases are a type of metalloenzyme with distinct characteristics, including stability across different solvents, surfactants, and bleaching agents, as well as compatibility with certain commercial detergents. These enzymes also exhibit thermal stability and excellent compatibility with some commercial liquid detergents [75].

##### Co-Culture Fermentation Strategies for Chitin Extraction

Co-culture systems, where multiple microbial strains are grown together, can offer significant advantages over single-strain (monoculture) systems due to the synergistic interactions among the different strains’ metabolic pathways. This synergy can lead to more efficient substrate use and improved production of target compounds. An additional benefit of co-culture is the ability to use secondary by-products, such as lactoserum, molasses, and other industrial processing waste, as cost-effective substrates for the production of various biotechnological products. This approach can help to reduce reliance on expensive sugars like glucose, making biological production processes more economically viable [76].

Moreover, co-culture systems can facilitate the discovery of novel compounds with industrial applications. During co-cultivation, a wider range of secondary metabolites is produced, potentially yielding substances of commercial interest [76].

The advantages of co-cultures are particularly noteworthy for the production of industrial products such as lactic acid and proteases, which are used to extract chitin from crustacean shell waste. In this context, lactic acid primarily contributes to demineralization by lowering pH and solubilizing calcium carbonate, whereas proteases facilitate deproteinization by hydrolyzing shell proteins closely associated with the chitin matrix. Lactic acid production typically involves fermentation with glucose or polymers that release glucose as the carbon source. For complex substrates containing cellulose or starch, the inclusion of hydrolytic enzymes such as cellulases and amylases has been shown to improve substrate accessibility and enhance overall chitin recovery efficiency [76]. However, using co-culture systems in simultaneous saccharification and fermentation processes can eliminate the need for these costly enzymes. This approach allows the use of high-concentration substrates, reducing production volumes and costs [76].

Low-cost carbon-rich substrates are especially valuable in lactic acid production, as the carbon source is typically the most significant expense in microbial production processes. Using co-culture fermentations can reduce costs while maintaining high productivity [63].

#### 2.2.3. Green Alternative Treatments

##### Deep Eutectic Solvents (DESs)

Deep eutectic solvents (DESs) were first reported by Abbott et al. [76] in 2003, who combined choline chloride with urea to yield a liquid mixture with unique solvent properties. DESs are sustainable, non-toxic, biodegradable, and have low vapor pressure, making them appealing for various applications, particularly for the extraction and processing of chitin [77,78].

DESs are a subclass of ionic liquids (ILs) composed of hydrogen bond donors (HBDs) and hydrogen bond acceptors (HBAs) [79] linked by hydrogen bonds [80]. When combined in specific molar ratios, HBDs and HBAs result in a lower melting point than the individual components. The most common HBA is choline chloride, a low-cost, biodegradable, biocompatible, and safe salt that can be industrially produced or extracted from biomass. Other HBAs used in choline-based DESs include choline acetate, choline bromide, choline dihydrogen citrate, choline hexafluorophosphate, and choline iodide [81]. These can be combined with urea or polybasic alcohol to create DESs with similar properties to those of choline chloride-based DESs.

Solvent recovery and recyclability are key factors supporting the sustainability of DES-based processes. In the literature, DESs have been successfully recovered and reused over several cycles with limited loss of extraction efficiency. Recovery is commonly achieved through anti-solvent addition (e.g., water), which reduces solvent–solute interactions and promotes precipitation of the extracted compounds, followed by solvent regeneration via evaporation. Other approaches include membrane-based separations and liquid–liquid extraction. Despite these promising results, challenges such as high viscosity, potential structural changes upon water uptake, and energy demand during solvent regeneration may affect recyclability, particularly at larger scales [82].

Although DES-based processes have demonstrated high efficiency at the laboratory scale, their industrial implementation is still at an early stage, mainly limited by challenges related to solvent recovery, viscosity, and large-scale process integration. However, their low cost and environmental compatibility make them promising candidates for future industrial applications [81].

##### Ionic Liquids (ILs) and Natural Deep Eutectic Solvents (NADESs)

The initial DESs contained metal ions. The need for lower toxicity prompted the development of natural deep eutectic solvents (NADESs), which are produced from primary plant metabolites, including sugars, organic acids, amino acids, alcohols, and amines. NADESs are generally classified into five types: ionic liquid NADESs, which consist of an acid and a base; neutral NADESs made of sugar and polyalcohol; neutral NADESs with bases, containing sugar/polyalcohol and organic bases; neutral NADESs with acids, composed of sugar/polyalcohol and organic acids; and amino acid-based NADESs, which contain amino acids and organic acids [83].

**Figure 5:**
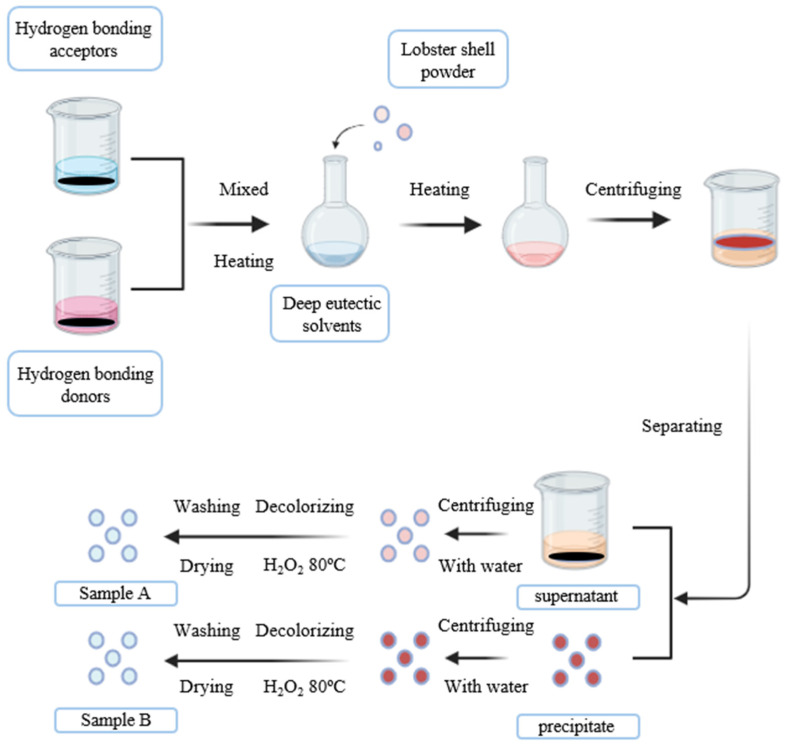
Extraction procedure of chitin by natural deep eutectic solvent (NADES).

NADESs have emerged as promising green alternatives to conventional organic solvents for chitin extraction [84]. Schematic illustration of the sequential extraction of chitin from shrimp shells by NADESs is shown in Figure 5.

The key characteristics of NADESs include their ability to carry out demineralization, deproteinization, and chitin dissolution in a single step. Chitin, which contains CaCO_3_, requires an acidic medium and an HBD cosolvent for extraction. Commonly used acidic solvents in this process include citric, malonic, and lactic acids. The mechanism involves NADESs breaking the strong hydrogen bonds between amino groups and H^+^ in chitin, thereby making them efficient dissolution media for hard cell wall polysaccharides such as chitin, cellulose, lignin, and starch. The NADES mixture comprises multiple solvents, usually two or three, each with a different melting point. This natural eutectic mixture is both environmentally safe and effective for extraction [85,86,87].

NADESs offer a green alternative to traditional solvents due to their sustainable characteristics: they are composed of simple, often naturally derived components that are readily sourced from basic chemicals. Comparative studies showed that NADESs can achieve polyphenol extraction efficiencies comparable to or exceeding those of conventional hydroalcoholic systems, while enhancing antioxidant capacity and bioactivity. Additionally, they offer low toxicity, non-flammability, and biodegradability, and enable operation under milder temperature conditions [88,89,90]. NADESs are non-volatile, which allows reuse and recycling; their synthesis typically requires minimal energy; they are generally considered safe; they can decompose without releasing significant toxic substances; they exhibit extraction capabilities comparable to those of conventional volatile organic solvents; they are typically stable at elevated temperatures; and they are generally non-flammable [83].

NADESs have shown considerable potential for reuse, further enhancing their green credentials. Studies report that NADESs can be recycled for multiple extraction cycles, often maintaining high efficiency and preserving the bioactivity of extracted compounds. Recovery strategies typically involve dilution with water or ethanol to facilitate product separation, followed by removing the added solvent to regenerate the NADES. However, repeated reuse may lead to the accumulation of impurities and slight decreases in extraction performance. Additionally, their high viscosity and strong intermolecular interactions can complicate solvent handling and recovery. Therefore, further optimization of recycling procedures and process integration is required to enable their efficient large-scale application [91].

Despite these advantages, NADES-based extraction processes are currently at a pilot or laboratory scale, and further optimization of process economics, solvent recycling, and scalability is required before widespread industrial adoption can be achieved [88,89,90].

##### Subcritical Water Extraction

**Figure 6:**
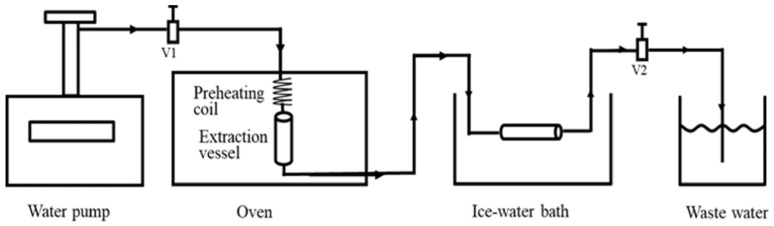
Schematic diagram of a subcritical water system [95].

Subcritical water extraction (SBWE), conducted at temperatures between 100 and 374 °C and under appropriate pressure, proved to be highly effective for producing chitin from various biomass sources, including agricultural residues and crustacean shells. The advantages of this method are the speed of the reactions and the use of a more sustainable solvent, replacing acids and bases [92,93,94]. Figure 6 shows a schematic diagram of an SBWE apparatus [95].

SBWE shows strong potential for industrial applications due to the use of water as the only solvent; however, its implementation is still limited by the need for high-pressure equipment and associated energy costs [95].

##### Ultrasonic-Assisted Extraction

The use of ultrasound enhances the solubility of proteins associated with chitin, facilitating their extraction. High-intensity ultrasound reduces the extraction time and eliminates the need for high temperatures. Pre-sonication treatment followed by steam explosion reduces the crystallinity of chitin. This method can be more effective and less destructive than conventional chitin extraction methods [96].

The strong dipole–dipole interactions in biopolymer chains influence the polymer’s crystallinity, making chitinase-mediated hydrolysis for chitin extraction more difficult. To overcome this obstacle, sonication and steam explosion are considered effective pretreatments [97].

Ultrasound-assisted extraction is already applied at an industrial scale in some sectors, although its integration into large-scale chitin production processes remains under development [96].

##### Integration of Electrochemical Extraction

Electrolysis, including the electrochemical treatment of crustacean exoskeletons to obtain chitin, was first reported by Kuprina et al. [98] and has emerged as an excellent alternative to chemical treatment.

**Figure 7:**
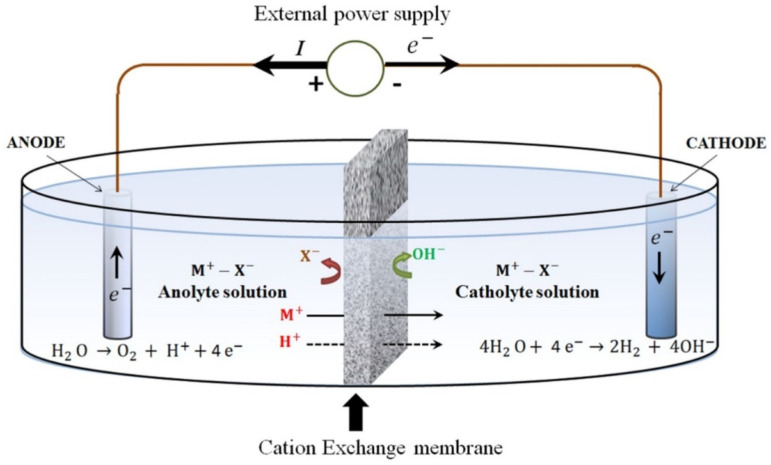
Schematic representation of an electrolyzer provided with an external power supply in the electrochemical application in chitin extraction [97].

The conventional electrochemical process involves an electrolyzer, or electrochemical cell, containing an aqueous solution of salts, typically composed of cations (M^+^) and anions (X^−^), where M+ represents alkali metal cations and X− represents anions (Figure 7) [97]. This electrolyte solution facilitates the transfer of ions. Two electrodes, an anode (negative) and a cathode (positive), constructed from chemically inert materials, commonly platinum, are immersed in the electrolyte. These electrodes have a high surface area and are separated by an ion-exchange membrane (either an anion, a cation, or a bipolar), dividing the electrolyzer into two compartments: the anode and cathode chambers. The low-molecular-weight salt solution acts as a conductive medium for the flow of electric charges [98].

Although electrochemical methods are attractive due to reduced chemical consumption, their industrial application is still emerging and depends on further optimization of energy efficiency and electrode material [98].

#### 2.2.4. Overview of Chitin Isolation Methods: Advantages and Limitations

**Figure 8:**
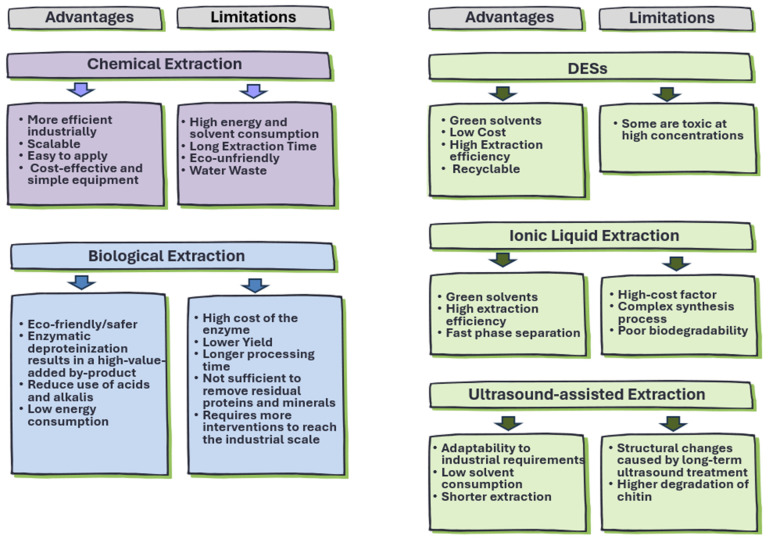
Advantages and limitations of the main methods used for chitin extraction.

As mentioned before, various methods have been successfully employed to extract and process chitin; however, these have not been adopted for scale-up or mainstream commercialization [99,100]. Each method has its own advantages and limitations, as summarized in Figure 8 [40,101]. Recently, there has been a growing effort to develop eco-friendly extraction processes based on green chemistry principles to reduce the use of hazardous compounds.

The selection of the most suitable method depends on the specific requirements and constraints of each application. Chemical extraction is more efficient for industrial-scale production; however, it is energy-intensive and generates significant waste, which is inconsistent with the principles of the green transition era. Biological extraction is environmentally friendly and safe, but it is associated with higher costs and longer processing times, highlighting the need for strategies to overcome these limitations. Ultrasound-assisted extraction offers good adaptability to industrial processes, representing a major benefit; however, it may lead to chitin degradation.

Despite the progress achieved with DESs for chitin extraction, their application remains at an early stage compared with conventional solvents. Further research is needed to develop strategies that enable their implementation on a commercial scale. The use of DESs for chitin extraction has only been explored since 2017, and many improvements are still needed. In addition, the relatively high cost of DESs and the challenge associated with their recovery and recycling remain major obstacles to their cost-effective industrial application.

### 2.3. Deacetylation of Chitin

Various methodologies can be employed to deacetylate chitin and produce chitosan. The deacetylation process not only modulates several physicochemical properties of chitosan, such as acid-base behavior, electrostatic characteristics, biodegradability, self-aggregation, solubility, sorption properties, and metal-ion chelation, but also determines its classification and suitability for distinct applications [102,103].

#### 2.3.1. Chemical Deacetylation of Chitin

Chemical deacetylation remains the dominant industrial route for chitosan production. This process involves complex treatments, which can result in significant waste and environmental pollution. Additionally, the chemical deacetylation process is challenging to control due to its random pattern [104].

Currently, chitosan is produced mainly by chemical deacetylation of chitin at an industrial scale because this route is cost-effective and suitable for high-throughput processing [79]. Conventional processes involve harsh treatments with concentrated alkali solutions at elevated temperatures, which facilitate *N*-deacetylation but also generate considerable quantities of alkaline effluent and solid residues, raising concerns about environmental pollution and waste management [99]. Alkaline deacetylation is preferred over acid deacetylation because glycosidic bonds in chitin are highly susceptible to acid. Chitin *N*-deacetylation can occur through heterogeneous or homogeneous processes [104].

In the heterogeneous method, chitin is treated with a hot (70–150 °C) 10–60% NaOH solution for several hours (typically around 6 h), yielding an insoluble chitosan fraction with a DD commonly in the range of 85–93%. DD increases with higher temperatures or NaOH concentrations [105]. In the homogeneous method, chitin is first dispersed in concentrated NaOH (approximately 13–24%) to prepare alkaline chitin, then dissolved in ice (0 °C); heterogeneous deacetylation produces soluble chitosan with a relatively low degree of deacetylation (approximately 48–55%) and distinct solution behavior [101]. The solubility of chitosan is influenced not only by the overall fraction of 2-acetamido-2-deoxy-D-glucose units but also by the distribution of *N*-acetyl groups along the polymer chain. An irregular sequence of *N*-acetyl-D-glucosamine and D-glucosamine residues, characteristic of heterogeneous deacetylation, strongly affects molecular aggregation and solution properties, thereby determining chitosan’s behavior in aqueous media [98,101]. Variations in processing parameters, including NaOH concentration, reaction time, temperature, and the number of successive alkaline treatments, can substantially modify average molecular weight, degree of acetylation, and viscosity, complicating reproducible control of product characteristics [104].

Deacetylation has been studied under a wide range of conditions. These include variations in the type of alkaline reagent (e.g., NaOH vs. KOH), reagent concentration, temperature, reaction time, and atmospheric environment. In addition, repeated alkaline treatments have been employed, while reducing agents such as NaBH_4_ are used to mitigate depolymerization. Kinetic and mathematical models have been developed to identify conditions required to achieve target degrees of acetylation, demonstrating the strong influence of temperature and alkaline reagent, with NaOH generally more effective than KOH. In addition, implementing controlled successive alkaline treatments and intermediate washing steps enables achieving high DD without prolonged single-step reactions, thereby improving process efficiency and minimizing polymer degradation.

Despite its advantages, namely industrial robustness and scalability, high reaction rate and DD, cost-effectiveness for bulk production, process flexibility, and tunable product properties, chemical deacetylation also presents several drawbacks [105]. The process requires a high energy input and large amounts of concentrated alkaline solutions, generating substantial waste streams and contributing to environmental pollution. Additionally, it produces significant quantities of both soluble and insoluble by-products. To address these limitations, alternative enzymatic methods have been developed [106].

#### 2.3.2. Enzymatic Deacetylation of Chitin

**Figure 9:**
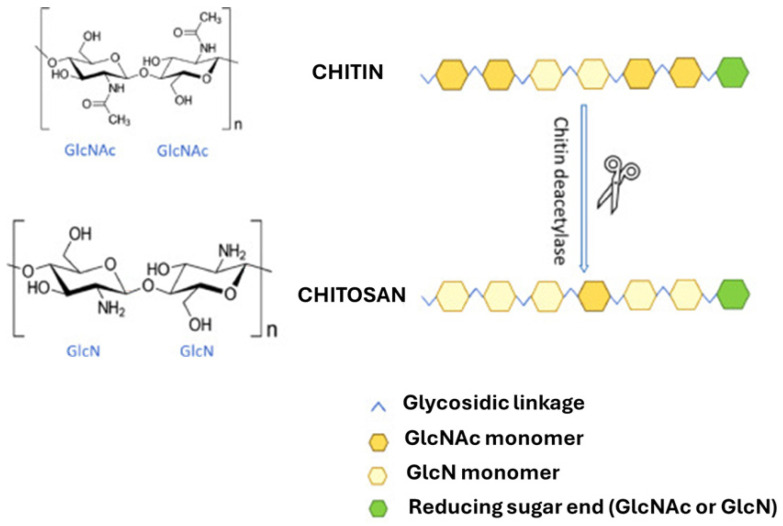
Conversion of chitin to chitosan by enzymatic deacetylation [107].

The enzymatic deacetylation of chitin is catalyzed by chitin deacetylases, as illustrated in Figure 9 [107]. These enzymes hydrolyze the acetamido group of *N*-acetylglucosamine, producing glucosamine and acetic acid. Consequently, the enzymatic method for chitosan production is gaining attention for its cleaner approach, higher efficiency, and greater specificity [108]. Chitin deacetylases enable regioselective, controlled deacetylation, allowing precise tuning of the degree and pattern of deacetylation, which is difficult to achieve with conventional chemical methods [109,110]. However, the kinetics of enzymatic deacetylation are generally slower than those of chemical processes, with reaction rates strongly dependent on substrate accessibility, crystallinity, and enzyme specificity. As a result, extended reaction times are often required, which can limit process productivity [111].

From a scalability perspective, several constraints remain. These include the cost of enzyme production, limited operational stability, and challenges associated with enzyme recovery and reuse. Although advances in recombinant expression systems have improved enzyme availability, economically viable large-scale implementation typically requires the development of efficient immobilization or recycling strategies [109]. In contrast, chemical deacetylation using concentrated alkaline solutions at elevated temperatures remains highly efficient, enabling high degrees of deacetylation within relatively short processing times, which explains its widespread industrial application despite its environmental drawbacks. From a techno-economic standpoint, enzymatic processes are currently less competitive than chemical methods due to lower space–time yields and higher catalyst costs. Nevertheless, they offer significant advantages, including milder operating conditions, lower energy requirements, and reduced environmental impact. These features make enzymatic deacetylation particularly attractive for sustainable processing and integrated biorefinery approaches [112].

Continued progress in enzyme engineering, process optimization, and reactor design is expected to improve the feasibility of enzymatic routes in the future. Chitosan deacetylases are mainly derived from bacteria, fungi, and insects [102].

##### Fungal Deacetylases

Fungal deacetylases can be divided into two groups based on their distribution in fungi. *Mucor rouxii* and *Agaricus coerulea* are located inside the cell’s periplasm and are called intracellular chitin deacetylases [113,114]. In contrast, *Colletotrichum lindemuthianum* and *Aspergillus nidulans* secrete chitin deacetylases into the surrounding culture medium and are therefore classified as extracellular chitin deacetylases. Extracellular deacetylases have significant biological relevance beyond mere chitin modification. In pathogenic fungi such as *C. lindemuthianum*, secretion of chitin deacetylase occurs specifically during hyphal penetration into host tissues, where it partially deacetylates exposed chitin in the cell wall, thereby reducing recognition by plant chitin-triggered immunity and facilitating infection and virulence. This deacetylation also influences the physicochemical properties of fungal cell walls, thereby enhancing resistance to plant chitinases secreted as part of the host defense response. In addition, extracellular chitin deacetylation can influence fungal morphogenesis, sporulation, and autolytic processes by modulating the composition of chitin and chitosan in the extracellular milieu [102,115].

These deacetylases are secreted at specific times that correspond to their biological functions. Different fungi secrete these enzymes at distinct developmental stages [116]. For example, an extracellular chitin deacetylase from *Colletotrichum lindemuthianum* is released only when fungal hyphae penetrate plant tissues, modifying chitin that would otherwise be recognized by the plant, and triggering a defense response. Conversely, an intracellular chitin deacetylase from *Mucor rouxii* [117] is produced during fungal cell wall formation. These enzymes are also produced during sporulation in *Saccharomyces cerevisiae* [99] and during vegetative growth in *Cryptococcus neoformans* [118].

Recently, a chitin deacetylase gene was found to be active during the development of fruiting bodies in the basidiomycete *Flammulina velutipes* [119].

##### Insect Deacetylases

Chitin deacetylases have been found in various insects, including *Anopheles gambiae* [120], *Apis mellifera* [121], *Drosophila melanogaster* [122], *Helicoverpa armigera* [123], *Mamestra configurata* [124], *Tribolium castaneum* [121], and *Trichoplusia ni* [125].

These enzymes are mostly located in the peritrophic membrane of the insect midgut, evenly spread throughout its length. For example, studies on *Trichoplusia ni*, *Helicoverpa armigera*, and *Mamaestra configurata* have demonstrated this distribution pattern [123]. Additionally, these enzymes are only present in the midgut tissue of larvae during their feeding period; they disappear from the tissue when the larvae stop feeding in later stages [125].

Although it is common for chitin deacetylases to be associated with the peritrophic membrane in insects, they are not limited to this location [125]. In *Drosophila melanogaster*, for instance, two proteins, chitin deacetylases CDA1 and CDA2 (known as serpentine and vermiform), were found in the tracheal extracellular matrix [120].

##### Marine Bacteria Deacetylases

*Vibrionaceae* are a family of marine bacteria widely distributed in oceanic and estuarine waters. They play a significant ecological role by degrading nitrogen-containing compounds present in chitinous debris that slowly sinks through the water column. This process contributes to recycling nutrients in marine environments [126].

In a study, Hunt and colleagues [127] conducted a comprehensive review examining the ecological and evolutionary dynamics of the *Vibrionaceae* family. Based on the analysis of genomes from 19 *Vibrio* and *Photobacterium* species, they proposed a pathway for chitin degradation. This pathway was compared with a previously assembled detailed metabolic map of Vibrio cholerae, using biochemical, genomic, and transcriptomic data.

To assess whether chitin degradation is a common trait among *Vibrionaceae*, the study evaluated 54 strains from 32 taxa for their ability to utilize various forms of chitin as a nutrient source. They found that all strains grew when provided with *N*-acetylglucosamine, a component of chitin. Most isolates also grew on chitin derived from crab shells and squid pens and possessed genes encoding chitinase A, an enzyme involved in chitin degradation. These findings indicate that chitin metabolism is a fundamental function shared across the *Vibrionaceae* family [126].

#### 2.3.3. The Most Recent Green Approaches

Traditional methods for chitosan production, particularly concentrated alkali treatments, have significant drawbacks, including high environmental impact and substantial financial investment for reagent purchases and neutralization of post-reaction solutions. These limitations hinder both scale-up and industrial implementation [128,129]. The recent trend is to develop approaches that employ more sustainable processes, minimizing environmental impacts on both the planet and industry, with emerging strategies establishing a new paradigm that embodies the principles of green and sustainable science [130].

One strategy to enhance the sustainability of chitosan production involves the reuse of chemical reagents. Zhao et al. [102] developed a method to recover NaOH from wastewater generated during chitosan production using stainless steel ultrafiltration and nanofiltration membranes. This method efficiently recovers NaOH, offering both environmental and economic benefits.

Energy consumption represents another critical factor in sustainable production. Conventional deacetylation processes typically require elevated temperatures; however, Nessa et al. [128] demonstrated effective chitin deacetylation at room temperature. This approach significantly reduced energy demand, offering clear cost savings and environmental benefits.

Another focus in improving existing methods is reducing waste, minimizing energy consumption, and avoiding harmful chemicals. The main goal is to develop industrial processes with minimal environmental footprint [131].

**Figure 10:**
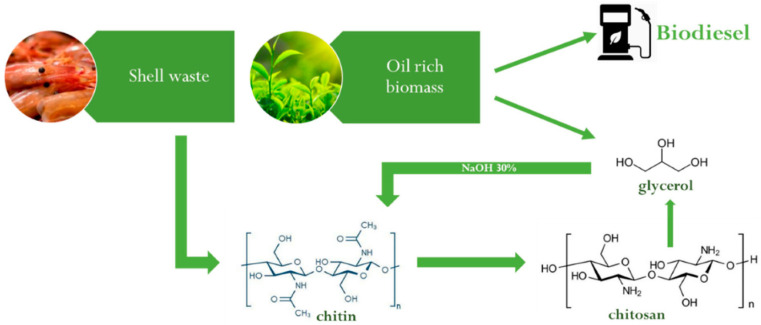
Optimized green procedure to deacetylate chitin in chitosan.

Sustainable production requires careful consideration of raw material use, energy demand, and waste generation at each stage. Liu et al. [118] proposed using glycerol, a recyclable and stable green solvent derived as a by-product of biodiesel production, as the reaction solvent (Figure 10). Operating at 180 °C, this method reduced the required NaOH concentration for chitin deacetylation, enhancing both process efficiency and environmental performance while exemplifying a circular economy approach.

The optimized procedure treated chitin with 30% NaOH and glycerol at a 1:40 chitin-to-glycerol ratio. To prevent glycerol polymerization in the alkaline environment, 1% water was added during the process [132].

This approach allowed chitosan production using lower NaOH concentrations and a valorized by-product, with both glycerol and NaOH recoverable and reusable in subsequent deacetylation reactions [118].

##### Deep Eutectic Solvents in Deacetylation

DESs have emerged as a sustainable and efficient alternative for producing chitosan from chitin. Their extensive hydrogen-bonding capability disrupts both intra- and intermolecular hydrogen-bond networks within chitin, facilitating its production. Although DESs can induce partial deacetylation of chitin, the DD under optimized conditions typically remains below 50%, the conventional threshold that distinguishes chitin from chitosan [133]. Sun et al. [133], following the disruption of chitin’s hydrogen-bonding network by the DES system, added a controlled amount of NaOH to further promote deacetylation. In addition, process parameters were optimized to obtain chitosan with an increased DD.

Overall, the incorporation of DESs not only enhanced deacetylation efficiency but also markedly reduced the required alkali concentration, decreasing environmental impact and providing a promising strategy for sustainable, industrial-scale chitosan production [126]. In a recent study using a NADES composed of betaine and glycerol, chitin was deacetylated to chitosan with a high DD (83.8%) using only 25 wt% NaOH at 100 °C for 12 h, whereas conventional alkaline deacetylation typically employs much higher NaOH concentrations of 40–50 wt% at elevated temperatures (often >100 °C) for comparable DD, representing approximately a 50% reduction in alkali concentration when using DES-assisted conditions. Traditional methods using concentrated NaOH not only require greater alkali input but also generate larger volumes of alkaline wastewater, increasing the burden on wastewater treatment systems and requiring neutralization before disposal. Although comprehensive data on energy consumption and waste volumes are still emerging, initial reports indicate that reduced alkali requirements in DES systems can lead to significantly lower volumes of alkaline waste liquor, improving process sustainability and decreasing environmental impact relative to conventional chemical deacetylation processes [40,133].

## 3. Modifications of Chitosan: Challenges and Opportunities

### 3.1. Classic Strategies

**Figure 11:**
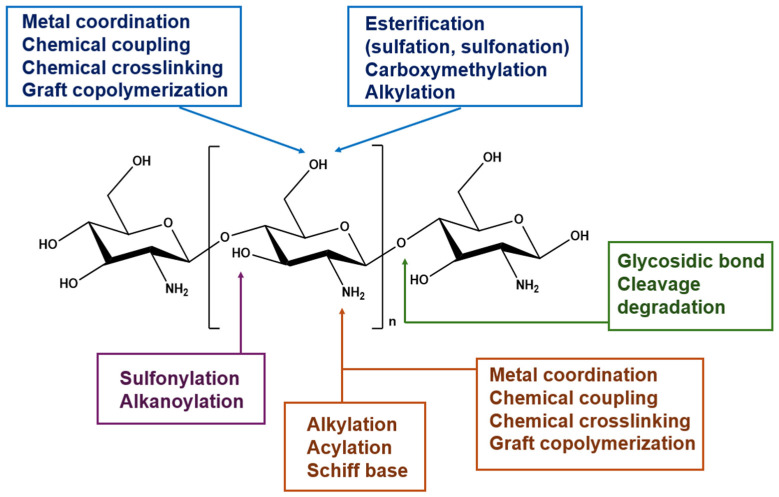
Functional groups in chitosan’s structure that can be chemically modified.

Chitosan contains reactive groups, including a primary amino group (C2) and primary and secondary hydroxyl groups (C6, C3), as well as glycosidic and acetamide moieties, providing diverse opportunities for chemical modification (Figure 11) and enabling the synthesis of polymers with tailored properties and functionalities [13].

Chemical transformations of chitosan include crosslinking, grafting, and partial degradation, leading to derivatives such as chemically crosslinked chitosan, grafted chitosan, low-molecular-weight chitosan, and oligochitosan, with improved solubility, stability, and bioactivity [134]. The amino groups undergo typical *N*-acylation and Schiff base formation, while the hydroxyl groups participate in *O*-acetylation and *O*-carboxymethylation reactions [135].

### 3.2. Green Strategies

To overcome the drawbacks of conventional derivatization, recent strategies prioritize green chemistry principles such as safer solvents, mild conditions, catalysis, and minimal waste [136]. In this context, click chemistry stands out as an attractive strategy for chitosan functionalization due to its modularity, high yield, selectivity, aqueous compatibility, benign by-products, and catalytic nature, all of which align with green chemistry principles [137].

#### 3.2.1. Click Chemistry

The term “click chemistry” was first introduced by K. B. Sharpless [138] in 2001 to describe chemical reactions ideal for quickly and reliably assembling chemical compounds by linking small units together. Click chemistry is a modular, biorthogonal approach adopted for the efficient synthesis of organic and bioorganic compounds [139,140]. In recent years, it has become a revolutionary tool for target research, leveraging its advantages of high specificity, biorthogonal compatibility, and modular design [138]. Typically, the reaction is fast and can be performed under simple conditions using readily available materials and reagents. Ideally, it should be solvent-free or employ harmless solvents such as water, and the product should be readily isolated without complex purification [141].

Click chemistry aligns with green chemistry principles and comprises a diverse set of reactions [142,143], with the most noticeable being the copper-catalyzed azide–alkyne cycloaddition (CuAAC) [144], the strain-promoted [3+2] azide–alkyne cycloaddition (SPAAC), the Diels–Alder reaction [145], and thiol–ene reaction [146].

Click chemistry represents an effective and greener alternative for obtaining chitosan derivatives [147,148], owing to its specific features that offer several significant advantages [149,150]. This type of reaction exhibits high stereoselectivity, regioselectivity, and chemoselectivity. In addition, it provides high yields, generates safe by-products (or none at all), and it is thermodynamically favorable, with a change in Gibbs free energy greater than 84 kJ mol^−1^, ensuring an efficient reaction process with high atom economy [151].

Polymer science is central to the creation of advanced functional materials, offering precise control over macromolecular architecture, composition, and properties. In recent years, click chemistry—especially azide–alkyne cycloaddition reactions—has become a highly versatile and efficient method for polymer synthesis, modification, and functionalization. Thanks to its exceptional efficiency, regioselectivity, and tolerance for diverse functional groups, azide-based click chemistry has greatly expanded the range of synthetic tools available to polymer researchers. Furthermore, its alignment with the principles of green and sustainable chemistry—such as high atom economy, mild reaction conditions, and minimal waste generation—makes it a cornerstone for the development of next-generation polymeric materials. As sustainability increasingly shapes the direction of materials science, integrating environmentally conscious methodologies into polymer click chemistry presents a promising route toward safer, more efficient, and resource-responsible polymer technologies [152].

For chitosan to participate in such reactions, it must first be chemically modified to introduce the appropriate functional groups, such as azide, alkyne, or thiol groups. Common modification strategies include: azidation of chitosan hydroxyl groups via reaction with sodium azide under acidic conditions; alkyne functionalization through coupling with propargyl bromide or propargyl amine in mild aqueous or organic media; and thiolation via reaction with thioglycolic acid or 2-iminothiolane. Reaction efficiency and degree of substitution typically depend on parameters such as reagent concentration, pH, temperature, and reaction time. These methods have been widely applied to produce bioorthogonally reactive chitosan derivatives suitable for click chemistry and other functionalizations [153,154]. Azide groups are commonly used in click chemistry due to their high reactivity with alkyne groups in azide–alkyne cycloaddition reactions. Alkyne groups react readily with azides in click reactions, forming stable triazole linkages. Highly dipolarophilic fragments can participate in [3+2] cycloaddition reactions. Thiol groups can participate in thiol–ene click reactions with double bonds. Double bonds can undergo click reactions with thiol groups, forming stable linkages. By incorporating these functional groups into chitosan, it becomes capable of participating in various click reactions, enabling the creation of complex, functionalized chitosan derivatives with potential applications in biomedicine, materials science, and other fields [139,142,155]. The application of click chemistry in polymer research has been emerging as a promising strategy for designing polymer-based hydrogels, drug and gene delivery systems, tissue-engineering scaffolds, and other advanced materials [156].

##### Copper-Catalyzed Azide–Alkyne Cycloaddition (CuAAC)

The CuAAC is the most well-known click reaction, involving the cycloaddition of an azide to a terminal alkyne in the presence of a copper catalyst under mild conditions [157]. It is highly efficient and yields triazole products with useful properties for many applications [158]. The CuAAC became particularly notable because it addressed many of the issues associated with the thermal version, such as reaction rate and temperature requirements [159,160,161]. Nevertheless, limitations include the cytotoxicity of copper catalysts, which limits their applicability in living systems, as well as the oxidative susceptibility of terminal alkynes, which require ligands to stabilize the catalytic copper center [162].

**Figure 12:**
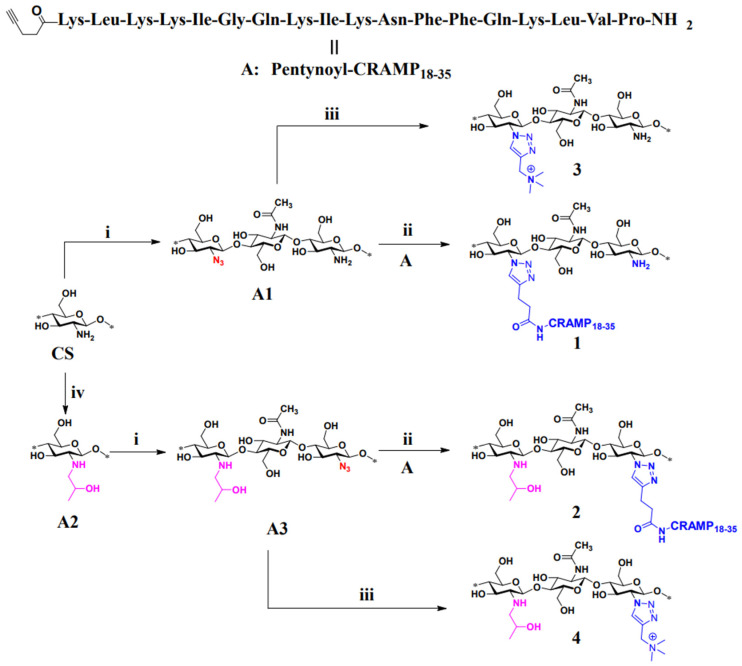
Conjugation of chitosan–CRAMP_18–35_ through diazo-transfer and subsequent click reaction; (i) 0.1 M HCl solution, sodium bicarbonate, imidazole sulfonyl azide HCl salt, CuSO_4_ 5H_2_O, water, methanol, RT; (ii) CuSO_4_ 5H_2_O, sodium ascorbate, pentynoyl–CRAMP_18–35_, DMSO, THPTA, 50 °C; (iii) CuSO_4_ 5H_2_O, sodium ascorbate, *N*-propargyl-*N,N,N*-trimethylammonium bromide, DMSO, 50 °C; (iv) propylene oxide, isopropyl alcohol, NaOH (30%). * asterisk bond for polymer chain linker continuation [164].

The practicality and reliability of CuAAC for chitosan modification were rapidly recognized, as evidenced by the following representative examples. A recent example of CuAAC involved the conjugation of a murine cathelicidin-related antimicrobial peptide (CRAMP_18–35_) carrying an *N*-terminal pentynoyl group to chitosan and hydroxypropyl chitosan azide (Figure 12) [163]. The chitosan–peptide conjugates displayed antibacterial activity, selectively targeting Gram-negative bacteria [163].

A simultaneous double-click reaction of azide–alkyne and amino-anhydride in water in one pot provided a facile method for the fabrication of an interpenetrating network hydrogel (IPN-Gel) drug carrier [165]. CuAAC between azide-modified chitosan (CS-ABA) and alkyne-modified sodium carboxymethylcellulose (CMC-PA) was performed to prepare PS-Gel. To create Cu(I), an in situ reduction reaction of CuSO_4_⋅5H_2_O and ascorbic acid (AA) was used. CC-Gel was formed by the amino-anhydride reaction between PMAN and ε-PLL. The swelling and drug-release behavior of the IPN-Gel were evaluated under different temperatures and pH values, revealing that it had outstanding thermal/pH dual responsiveness and biocompatibility [165].

**Figure 13:**
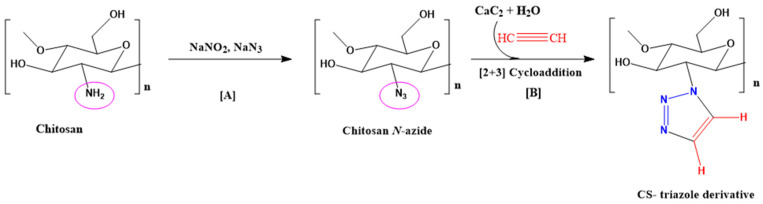
[A] Synthesis of chitosan *N*-azide. [B] Synthesis of chitosan-1,2,3-triazole derivative (CS-triazole derivative).

In another work, chitosan has been modified via CuAAC reaction to synthesize a chitosan-1,2,3-triazole derivative starting from CaC_2_. The chitosan-triazole derivative exhibited antibacterial activity against both Gram-negative (*E. coli*) and Gram-positive (*B. subtilis*) bacteria, as well as significant antioxidant properties (Figure 13) [164].

**Figure 14:**
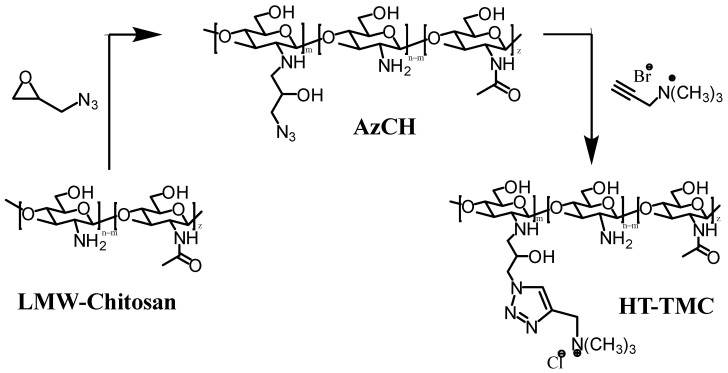
Synthesis of: *N*-(3-azido-2-hydroxypropyl) chitosan (AzCH) and *N*-[(2-hydroxypropyl-1,2,3-triazol-4-yl)-*N*,*N*,*N*-trimethylmethanammonium]chitosan chloride (HT-TMC). LMW-Chitosan: low-molecular-weight chitosan.

Under ultrasound-promoted CuAAC, new quaternized chitosan derivatives containing a 1,2,3-triazole fragment were obtained (Figure 14) [166]. First, *N*-(3-azido-2-hydroxypropyl) chitosan (AzCH) derivatives with different degrees of substitution were synthesized. The synthesis was carried out by forming Cu(I) in situ under ultrasound in aerobic conditions, in the presence of acetic acid and metallic copper (copper turnings) [167].

The introduction of a quaternary ammonium moiety led to new charged chitosan derivatives with enhanced antibacterial activity (against both Gram-positive and Gram-negative microorganisms), improved fungicidal properties, and increased water solubility across a broad pH range (3–12) [166].

##### Strain-Promoted [3+2] Azide–Alkyne Cycloaddition (SPAAC)

A metal-free alternative, known as SPAAC, exploits the inherent ring strain of cyclooctyne molecules to drive cycloaddition with azides, generating 1,5-disubstituted triazoles and effectively lowering the activation barrier [168]. Importantly, this strategy has been demonstrated to proceed efficiently in living systems without the need for cytotoxic copper catalysts, to label under mild reaction conditions, and to exhibit superior biocompatibility [169]. In the original report, biomolecular labeling was successfully achieved in living cells with no observable adverse effects on cellular function [168]. Subsequent studies have provided quantitative evidence of its biocompatibility; for example, cell viability assays in HeLa cells treated with cyclooctyne reagents showed viability levels above 90%, indicating minimal cytotoxicity under typical reaction conditions. These results confirm that SPAAC reactions can be performed in biological environments while preserving cell viability and function. Nonetheless, SPAAC is hindered by slow reaction kinetics and is less suitable for high-throughput workflows due to the expensive synthesis and limited stability of cyclooctynes [170].

This innovative approach was originally pioneered by the Bertozzi group [168], but, to further enhance reaction efficiency, extensive research has focused on developing highly reactive cyclooctyne reagents and analogs [171,172]. Strategies such as introducing electron-withdrawing substituents or increasing the intrinsic ring strain of cyclooctynes have successfully yielded activated reagents with reaction rates comparable to those of CuAAC. These advances have substantially expanded the utility of SPAAC and paved the way for its broader application across diverse scientific and industrial contexts, focusing on its remarkable selectivity, biorthogonality, and satisfactory reactivity under mild reaction conditions [147,173,174,175]. In polymer and materials science, it has rapidly gained recognition as a robust and environmentally benign coupling strategy, as evidenced by the following representative examples.

Recently, amphiphilic chitosan derivatives as drug carriers were synthesized by a modular strategy based on a SPAAC click reaction through tuning the hydrophobic groups. Through this strategy, *N*-azido propionyl-*N*,*O*-sulfate chitosan (ASC) reacted with azadibenzocyclooctyne-modified deoxycholic acid (ADIBO-DOC) or azadibenzocyclooctyne-modified octanoic acid (ADIBO-Oct) via SPAAC under gentle conditions to facilely afford deoxycholic acid- or octanoic acid-modified *N*-azido propionyl-*N*,*O*-sulfate chitosan (DCSC or OCSC, respectively). The modular strategy exhibited advantages including high reactivity, flexibility, and reproducibility [176].

The amphiphilic chitosan derivatives, DCSC and OCSC, served as drug carriers for encapsulating paclitaxel (PTX), a mitotic inhibitor used in cancer chemotherapy. The chitosan-based micelles exhibited high drug-loading capacity and good biocompatibility, highlighting their potential for in vivo use in cancer therapy. SPAAC reaction between azide-modified proteins and chitosan-poly(ethylene glycol) microparticles activated with strain-promoted cyclooctynes allowed tunable protein conjugation under mild reaction conditions. Initially, chitosan-PEG microparticles were produced via replica molding from a pre-particle solution containing chitosan and poly(ethylene glycol) diacrylate (PEGDA), yielding particles with typical diameters of 50–200 µm. The crosslinking density was controlled by the PEGDA content (5–15 wt%), which influenced both the mechanical stability and swelling properties of the microparticles [177]. For surface functionalization, the microparticles were activated with azadibenzocyclooctyne (ADIBO) groups in aqueous medium at room temperature (pH 7.0–7.4) for 2–6 h, ensuring efficient incorporation of strained alkyne functionalities. Azide-modified proteins were subsequently added at concentrations of 0.1–1.0 mg·mL^−1^ to yield stable triazole linkages. Under these conditions, coupling efficiencies in the range of 70–90% were achieved, depending on protein accessibility and surface functional group availability [177]. The synthetic strategy proved to be a feasible and efficient approach for producing protein-conjugated chitosan–poly(ethylene glycol) biosensing platforms for protein sensing applications and for in-depth evaluation of protein–particle conjugation kinetics [177].

##### Diels–Alder Reaction

The Diels–Alder reaction was discovered by Otto Diels and Kurt Alder [178] in 1928, for which they were awarded the Nobel Prize in Chemistry in 1950. A typical [4+2] cycloaddition Diels–Alder reaction involves a conjugated diene, typically electron-rich (such as furan), and an electron-poor dienophile (often an alkene, like maleic acid), generating a highly selective and regiospecific (substituted) cyclohexene system [148].

The reaction is characterized by its modularity, broad scope, high yields, and generation of minimal or non-toxic by-products [179]. Typically, it proceeds under mild reaction conditions and is thermally reversible, allowing control over the reaction extent [180,181]. Moreover, it is generally carried out without the need for catalysts or initiators, thereby preserving the material’s biocompatibility [182]. Its versatility and efficiency have contributed to its widespread use in organic chemistry as well as polymer and materials science [144,183,184,185]. Representative examples are presented below.

Recently, a Diels–Alder click reaction between *N*-furfuryl chitosan and various poly(ethylene)glycol-maleimide derivatives in dilute aqueous acidic conditions afforded clear, transparent hydrogels with excellent mechanical properties [186].

**Figure 15:**
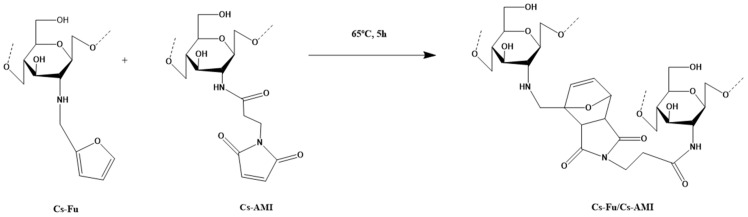
Diels–Alder reaction between furan-functionalized chitosan (Cs-Fu) and maleimide-functionalized chitosan (Cs-AMI).

A chemically crosslinked chitosan-based hydrogel was successfully synthesized via a Diels–Alder reaction between two complementary chitosan derivatives, namely furan-functionalized chitosan (Cs-Fu) and maleimide-functionalized chitosan (Cs-AMI) (Figure 15). The two chitosan derivatives were cross-linked to the final hydrogel network. The hydrogel exhibited pH-responsive behavior, biocompatibility, and antibacterial activity, all of which are promising characteristics for biomedical applications, particularly in targeted drug delivery [187].

In another study, Cs-Fu was also used as a precursor to prepare stimuli-responsive chitosan-based hydrogels using the Diels–Alder reaction. In this case, Cs-Fu was crosslinked with polyetheramine-derived bismaleimide (BMI). The chitosan-based hydrogels exhibited promising characteristics as potential carriers for targeted drug delivery [188].

##### Thiol–Ene Click Reaction

Among metal-free click reactions, the thiol–ene click reaction stands out for its proven efficiency and versatility. This click reaction displays remarkable tolerance toward ambient oxygen and moisture, affords a single regioselective product, and can be readily applied across a broad spectrum of thiols and alkenes [146]. The thiol–ene reaction is radical-based, involving the attack of a thiol radical on an electron-rich or electron-poor carbon–carbon double bond to form C-S-C linked adducts, and it can be initiated photochemically or through other free-radical initiators [138,189].

One limitation of thiol-containing reactions is their tendency to undergo oxidation, forming disulfide bonds, a challenge that is particularly pronounced with high-molecular-weight thiolated reagents such as thiolated polysaccharides [190]. Careful preparation and storage, including the use of antioxidants or inert atmospheres, are therefore required to minimize oxidative degradation [191]. In addition, small-molecule thiols may pose odor and toxicity issues, hindering large-scale industrial implementation. The exceptional versatility and efficiency of the thiol–ene click reaction, together with its propensity for near-quantitative conversions under mild conditions, make thiol–ene chemistry highly suitable for polysaccharide modification [138,189].

In the context of magnetic mesoporous silica/chitosan (MMS/CS) systems, thiol–ene functionalization enables the controlled introduction of reactive groups that significantly enhance material performance. For example, the incorporation of aldehyde functionalities via thiol–ene chemistry allows subsequent covalent grafting of chitosan, leading to increased functional group density and improved adsorption behavior. Similar systems reported in the literature exhibit high adsorption capacities for heavy metal ions, typically in the range of 120–180 mg·g^−1^, which is attributed to the synergistic combination of mesoporosity and abundant amine binding sites. Although surface area may decrease after functionalization, the increased surface reactivity generally results in enhanced affinity and selectivity toward target species. Furthermore, thiol–ene-modified MMS/CS composites have been shown to possess good structural stability and reusability, often retaining more than 80% of their initial adsorption capacity after several adsorption–desorption cycles. These findings demonstrate that thiol–ene modification is not only an efficient functionalization strategy but also a key factor in improving the physicochemical properties and application performance of polysaccharide-based hybrid materials [192]. Other representative examples are presented below.

**Figure 16:**
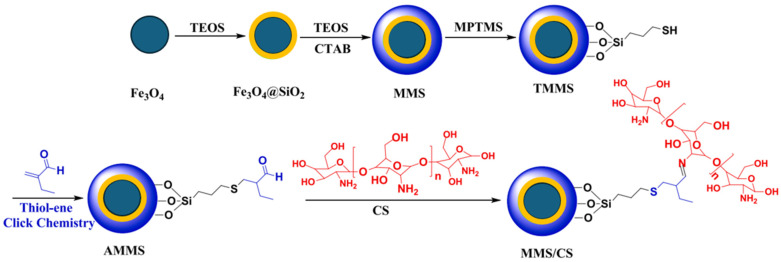
Schematic illustration of the fabrication process for a magnetic mesoporous silica/chitosan (MMS/CS) composite via thiol–ene click reaction. AMMS: aldehyde group-modified MMS; CTAB: cetrimonium bromide; MPTMS: 3-mercaptopropyltrimethoxysilane; TEOS: tetraethyl tetraethoxysilane; TMMS: thiol-functionalized MMS.

Recently, thiol–ene click chemistry enabled the development of novel MMS/CS composites as multifunctional bioadsorbents for selective capture of toxic Hg(II) from aqueous media and subsequent utilization as an efficient heterogeneous catalyst to reuse the spent Hg(II) adsorbent [192]. As illustrated in Figure 16, the first step was the preparation of thiol-functionalized MMS (TMMS) by using Fe_3_O_4_ nanoparticles as the magnetic core, which was then coated with mesoporous silica and further functionalized with (3-mercaptopropyl)triethoxysilane (TEOS) [192]. Then, the TMMS was subjected to a click reaction with 2-ethylacrylaldehyde to yield aldehyde-modified MMS (AMMS). The unmodified TMMS exhibited a specific surface area of approximately 850 m^2^·g^−1^, which decreased to approximately 620 m^2^·g^−1^ after aldehyde functionalization (AMMS), indicating successful surface modification and partial pore occupation. Upon formation of the MMS/CS composite, the surface area further decreased to approximately 410 m^2^·g^−1^, consistent with the incorporation of chitosan into the mesoporous structure. Finally, MMS/CS was synthesized by reacting AMMS with CS via a Schiff base condensation.The functional group density of AMMS was ~1.26 mmol·g^−1^ (aldehyde groups). After reaction with chitosan, MMS/CS exhibited an amine density of ~0.97 mmol·g^−1^, confirming successful grafting onto the mesoporous silica surface. Compared with the conventional crosslinking approach, the proposed thiol–ene click chemistry strategy enables the synthesis of MMS/CS with a relatively larger surface area and a higher density of active functional groups (amino and thioether) [192].

Another recent work described the preparation of a nanocomposite hydrogel based on a hydrophilic chitosan derivative and chemically modified graphene oxide via a thiol–ene click reaction for human motion sensing [193]. Thiol-functionalized graphene oxide [176] nanosheets served as a chemical crosslinker for hydrogel formation through a thiol–ene click reaction between methacrylated zwitterionic chitosan and nanosheets. The novel hydrogel demonstrated rapid self-healing and UV light-induced gelation at room temperature, and exhibited degradability, biocompatibility, conductivity, and multifunctionality [193].

**Figure 17:**
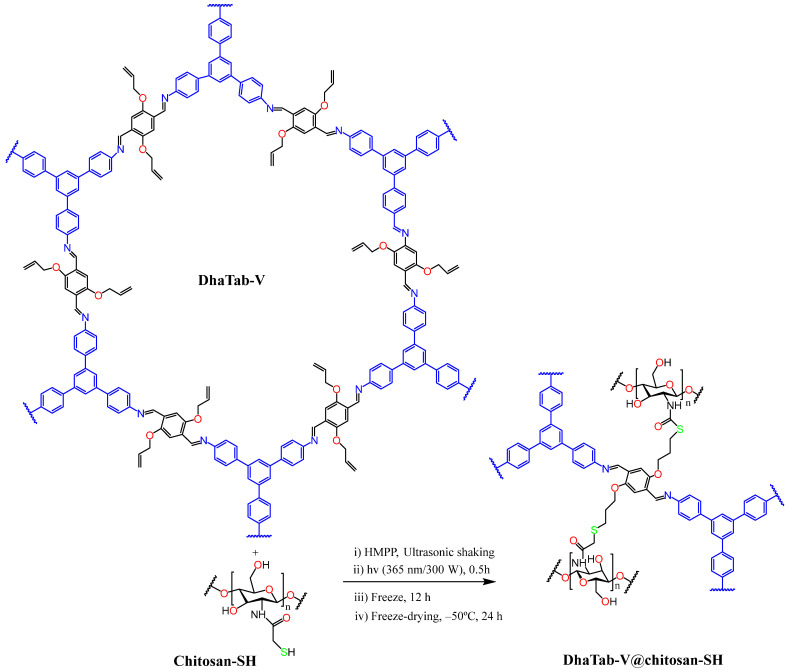
Synthesis process of chitosan-modified covalent organic framework (COF) material (DhaTab-V@chitosan-SH) through a photoinduced thiol–ene click reaction between the precursor COF (DhaTab-V) and thiol-functionalized chitosan (chitosan-SH). HMPP: 2-hydroxy-2-methylacetone.

In another study, a novel chitosan-modified covalent organic framework (COF) (DhaTab-V@chitosan-SH) was prepared via a photoinduced thiol–ene click reaction for the efficient and selective recovery of gold from complex liquids [194]. Adopting the photo-induced thiol–ene click reaction, the precursor COF (DhaTab-V) rich in vinyl groups was efficiently crosslinked with thiol-functionalized chitosan (chitosan-SH) (Figure 17) [194].

Recently, other thiol-functionalized chitosan derivatives have been employed to generate chitosan-based materials via thiol–ene click chemistry for various applications. For example, for the development of cisplatin-loaded folic acid-decorated nanoparticles as a targeted drug carrier in oral carcinoma, folic acid-conjugated thiolated succinyl chitosan (FA-SH-SCS) was synthesized. Then, FA-SH-SCS reacted with maleimide-grafted-carboxymethyl cellulose (CMC-MAL) via thiol–maleimide click reaction, yielding the folic acid–decorated nanoparticles (FA-NPs) [195]. FANPs enhanced the cellular uptake of cisplatin in oral carcinoma cells by specifically recognizing folate receptors. The delivery of cisplatin by FA-NPs to KB cells induced apoptotic cell death. The FA-NPs exhibited an average particle size of 145 ± 12 nm, a positive zeta potential of +21.3 ± 2.1 mV, an encapsulation efficiency of 87.5 ± 3.2%, and a drug loading of 9.8 ± 0.6%, which contributed to their effective cellular uptake and cytotoxicity [195].

**Figure 18:**
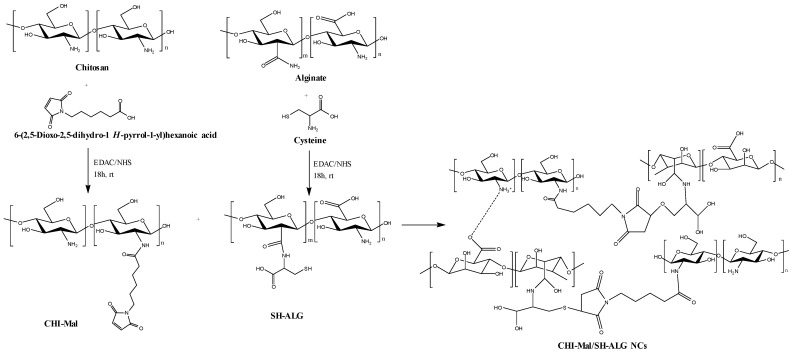
Synthesis of chitosan-maleimide (CHI-Mal) and thiolated alginate (SH-ALG) followed by thiol–maleimide click reaction to prepare bioconjugated clicked chitosan/alginate nanocarriers (CHI-Mal/SH-ALG NCs). EDAC: 1-ethyl-3-(3-dimethylaminopropyl)carbodiimide; NHS: *N*-hydroxysuccinimide.

Furthermore, using thiol–maleimide click chemistry, bioconjugated clicked chitosan/alginate nanocarriers (CSMal/SH-ALG NCs) were engineered with trastuzumab, a potent antibody targeting HER2-positive breast cancer cells, for curcumin delivery to targeted tumor cells. Chitosan-maleimide (CHI-Mal) and thiolated alginate (SH-ALG) were synthesized to produce the CSMal/SH-ALG NCs, which were subsequently conjugated with trastuzumab as a receptor-targeting ligand via click chemistry (Figure 18) [196]. Curcumin was encapsulated via entrapment, resulting in trastuzumab-conjugated curcumin-nanocarriers (Tras-Cur-NCs) with a particle size of 162 ± 15 nm, a zeta potential of +18.7 ± 1.9 mV, an encapsulation efficiency of 91.2 ± 2.7%, and a drug loading of 11.4 ± 0.8%. These characteristics enabled enhanced anticancer activity, improved receptor-targeting specificity, and greater cellular uptake compared with free curcumin. Moreover, Tras-Cur-NCs significantly promoted apoptotic cell death [196].

## 4. Applications for Chitosan and Its Derivatives

**Figure 19:**
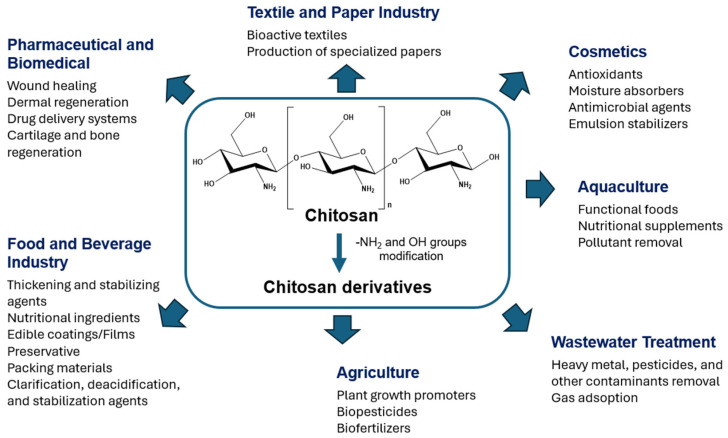
Main areas of application of chitosan and its derivatives.

Chitosan and its derivatives are used in a wide range of sectors, from agriculture and food production to pharmaceuticals and biomedical applications, owing to their unique properties and multifunctional capabilities. Figure 19 summarizes the major applications for chitosan and its derivatives [197,198].

The diversity and continuously expanding range of these applications are key drivers of growth in the global market for these polysaccharides. Representative examples from the main application areas are discussed below.

### 4.1. Pharmaceutical and Biomedical Industry

Chitosan, a non-toxic and biodegradable polysaccharide with diverse biological activities, is widely recognized for its potential in a broad range of biomedical applications [197,199]. Its versatility arises from the possibility of chemical modification, enabling the development of derivatives with enhanced antioxidant, antimicrobial, antiviral, and antitumor properties, among others [200,201].

For instance, chitosan derivatives such as carboxymethyl chitosan thiosemicarbazones can delay radical formation by acting as hydrogen donors, thereby preventing the propagation of oxidative chain reactions [202,203]. Similarly, selenourea-functionalized chitosan derivatives have demonstrated high antioxidant activity [204].

Several modified forms also exhibit strong antimicrobial properties. The quaternary derivative *O*-xanthonyl-chitosan [205] and Schiff base derivatives of chitosan [206] showed significant antibacterial activity, while propane-sulfonated and dipropane-sulfonated chitosan derivatives displayed notable antifungal effects [207]. In addition, *O*-acyl chitosan derivatives were reported to possess both fungicidal and insecticidal activity [208].

Chitosan and its derivatives also present relevant antiviral properties [209]. Sulfated and sulfonated derivatives have been shown to inhibit the human immunodeficiency virus (HIV) [210]. Moreover, potential activity against the coronavirus SARS-CoV-2 has been explored [211]. In particular, *N*-benzyl-*O*-acetyl-chitosan, imino-chitosan, and sulfated derivatives exhibited high affinity for the receptor-binding domain of the spike protein of SARS-CoV-2 [212]. More recently, selenium nanoparticles produced via green biosynthesis using *Limosilactobacillus fermentum*, by incubating the bacterial supernatant with sodium selenite under mild pH and temperature conditions, and subsequently functionalizing with chitosan, were reported to effectively inhibit SARS-CoV-2. Chitosan coating enhanced nanoparticle stability and antiviral activity, achieving significant inhibition of viral replication [213].

Beyond therapeutic applications, chitosan-based materials have also been investigated as antiviral disinfectants. A hydrogel composed of partially acrylated chitosan combined with organic or inorganic components has been developed and patented, which can be used as an antiviral spray or as liquid antiviral gloves [214].

In addition, chitosan has demonstrated the ability to inhibit tumor cell growth without significant toxicity toward non-tumor cells [215]. Among its derivatives, the chitosan–thymine conjugate represents a notable example of a compound with promising antitumor activity [216].

One of chitosan’s key biomedical uses is in wound healing [217,218]. We can find many chitosan-based products on the market that serve as topical dressings for treating wounds. The addition of chitosan and its derivatives to bandages enhances their effectiveness due to their antimicrobial properties and biocompatibility [219]. They also exhibit a cell-stimulating effect, accelerating wound healing and dermal regeneration. In wound care products, chitosan’s inherent antimicrobial and hemostatic properties are combined with its drug-delivery capacity, thereby facilitating tissue growth and regeneration [220,221].

In the realm of tissue engineering, which seeks to restore, improve, or replace various biological tissues, chitosan and derivatives are used to create scaffolds with excellent controlled-release properties for delivering therapies and growth factors. They are employed in tissue engineering to create biomaterials for cartilage, bone, blood vessels, skin, periodontal tissue, and corneal regeneration [222]. The mechanical strength and structural integrity of chitosan-based biomaterials can be enhanced by incorporating other biopolymers, such as chitin, alginate, polylactic acid, hydroxyapatite, and bioactive nanoceramics [131,223].

Chitosan and its derivatives are also valued for various drug delivery systems, including oral, parenteral, and topical administration, as well as targeted drug delivery [224,225,226]. Additionally, they have crosslinking capabilities with other polymers, antimicrobial properties, gel-forming ability, bioadhesion, immunostimulatory effects, macrophage activation, and gas permeability [227]. Chitosan-based systems are used for the delivery of drugs [228,229,230,231], proteins [232,233], genes [234,235], vaccines [236,237,238], siRNA therapeutics [239,240,241], among others.

### 4.2. Cosmetics

Lately, there has been growing interest in cosmetic products that also have health benefits. Chitosan possesses a unique set of biological properties that make it an attractive candidate for the development of cosmetic and therapeutic formulations to treat dermatological conditions and modulate the skin microbiome [242]. Chitosan proved to be an ideal candidate for encapsulating essential oils, either alone or in combination with other polymers, being an effective encapsulating agent that protects biomolecules from environmental and processing factors [243]. Chitosan-based cosmetics with pharmaceutical or medicinal properties are already available on the market. They often contain essential oils and active ingredients like enzymes, antioxidants, vitamins, and phytochemicals. These products are available as creams, lotions, and ointments [244,245].

For example, carboxymethyl chitosan derivatives can be used as multifunctional and biocompatible ingredients in cosmetic formulations, serving as antioxidants, humectants, antimicrobial agents, and emulsion stabilizers [245]. Another example is the use of thiadiazole-chitosan derivatives as a novel cosmetic ingredient in rinse-off hair conditioners [246]. Additionally, chitosan-based active packaging has proven to be a promising innovation strategy in the cosmetic sector [246,247].

### 4.3. Agriculture and Aquaculture

Chitosan and derivatives have been employed in agriculture since the 1990s as bactericides and bacteriostatics to protect plants from pathogenic bacteria that can damage crops during both the growing and post-harvest stages. These biopolymers activate plant defense mechanisms, promote the accumulation of secondary metabolites, and, in turn, boost the plant’s resistance to disease. Chitosan’s chelating properties also make it useful in sprays designed to remove pesticides, and it is an effective antifungal agent. Incorporating chitinous biomass into soil can further enhance crop protection by stimulating beneficial microbes, particularly *Bacillus* spp. Additionally, chitosan derivatives can be used for the controlled release of pesticides and herbicides [248,249].

Chitosan and its derivatives also play a key role in aquaculture, acting as functional feed additives, nutritional supplements, and carriers for bioactive compounds, and encapsulating pathogens or nucleic acids, and for removing pollutants from wastewater. For example, chitosan-based encapsulation systems have been reported to improve the stability and delivery efficiency of nutrients, probiotics, and vitamins, with encapsulation efficiencies of typically 70–90%, depending on the formulation and preparation method. Furthermore, chitosan nanoparticles and microcapsules have been widely explored for the delivery of bioactive molecules, thereby enhancing bioavailability and biological activity [146]. In aquaculture water treatment, chitosan exhibits excellent flocculation and adsorption properties, with removal efficiencies frequently exceeding 80% for suspended solids and significant reductions in contaminants, thereby improving water quality and system sustainability [248,249]. As a result, chitosan has been successfully integrated into seafood products to improve food quality, stability, and nutritional content [146].

### 4.4. Wastewater Treatment

Growing environmental awareness and the pressing issue of water pollution caused by heavy metals, pesticides, and other contaminants have spurred the search for new methods to purify water before it is released into the environment. Chitosan and its derivatives are commonly used in wastewater and effluent treatment because their amino groups can bind metal ions, allowing efficient removal of contaminants [250,251]. For example, chitosan, carboxymethyl chitosan, and crosslinked chitosan have been shown to be effective at removing heavy metal ions such as Ni^2+^, Co^2+^, and Cu^2+^ from drinking water [252].

Moreover, the paper industry is one of the most polluting sectors due to its intensive use of chemicals. Chitosan, with its high cationic charge density and long polymer chains, exhibits strong flocculating and coagulating abilities, making it a promising, environmentally friendly alternative for paper mill wastewater treatment [253]. Additionally, chitosan can absorb dyes, humic acids, metal ions, and bacterial and xenobiotic cells from wastewater generated by paper production and other industries [254,255].

### 4.5. Food and Beverage Industry

In the food industry, chitosan and its derivatives are used as low-cost natural thickening and stabilizing agents in processed foods. Due to their bioactive properties and cationic nature, they also serve as nutritional ingredients and as antimicrobial and antioxidant agents in food production, preventing microbial spoilage, extending the shelf life of food products, creating biodegradable films, and improving food packaging [256,257].

Chitosan is also applied as a coating for fruits to reduce moisture loss and delay ripening, offering a biodegradable alternative to more toxic polymers. This effect is attributed to the selective gas permeability of chitosan films [258]. Additionally, chitosan and its derivatives are widely used in functional food packaging, including paper-based packaging and new types of functional packaging films that incorporate various plant extracts and essential oils, like prickly pear extract, lemon essential oil, and *Eucalyptus globulus* essential oil [259].

In the beverage industry, chitosan and derivatives are particularly useful in wine production for clarification, deacidification, and stabilization. These polymers have been effective in removing ochratoxin, a mycotoxin produced by certain fungi during the winemaking process, which can cause acute kidney toxicity in mammals and is carcinogenic to humans. Moreover, they are also used in brewing as natural flocculants to clarify beverages [260].

### 4.6. Textile and Paper Industry

Chitosan is used to enhance the paper manufacturing process due to its antibacterial properties and film-forming ability, enabling the production of specialized papers, such as antibacterial and oiled papers. Additionally, using chitosan as an additive in papermaking has improved various paper characteristics, including dry and wet strength, color retention capacity, and effectiveness as a retention and drainage aid [261,262].

Due to its antimicrobial, biodegradable, biocompatible, and non-toxic properties, chitosan has become an attractive material for textile applications, enabling the development of bioactive textiles. It can act as a bioactive finishing agent, imparting antimicrobial properties to conventional textiles and cosmetotextiles. Additionally, chitosan offers several functional advantages in textile processing, including antistatic and deodorizing effects, metal-chelating ability, film-forming capacity, chemical reactivity, cost-effectiveness, and the potential to enhance dyeing performance [263].

## 5. Conclusions

This review highlights the significant potential of chitosan and its derivatives in the context of the green transition era, emphasizing sustainable production routes and environmentally friendly applications. The main sources of chitin, including crustaceans, fungi, and insects, as well as extraction, deacetylation, and modification strategies, were critically discussed, with particular emphasis on the implementation of green chemistry principles.

Conventional chemical methods remain advantageous for efficiency and control over polymer properties; however, their environmental impact and cost remain significant drawbacks. In contrast, biological and emerging green approaches, including the use of deep eutectic solvents and ultrasound-assisted extraction, offer more sustainable alternatives, although challenges related to scalability, efficiency, and process optimization persist.

Modification strategies, particularly those based on click chemistry, offer powerful tools for the design of functional chitosan derivatives with tailored properties. These approaches enable precise functionalization, expanding the applicability of chitosan-based materials across diverse sectors.

Despite these advances, several limitations must be addressed to enable large-scale implementation. These include variability in chitosan quality depending on raw material sources and processing conditions, difficulties in solvent recovery and recycling, potential toxicity associated with certain reagents and modifications, limited reproducibility, and regulatory constraints. Overcoming these bottlenecks will require standardized protocols and improved process control.

Future research should focus on the development of scalable, reproducible green extraction and modification processes, as well as on the implementation of sustainability assessment tools such as life-cycle analysis, techno-economic analysis, and circularity metrics. In addition, process intensification strategies, including continuous processing and improved solvent management, should be prioritized to enhance industrial feasibility.

Opportunities for further advancement include the integration of chitosan production into biorefinery systems and the valorization of waste biomass streams, which may significantly improve resource efficiency and sustainability. Interdisciplinary approaches will be essential to bridge the gap between laboratory-scale developments and industrial applications.

Overall, the combination of functional versatility and biological activity, including antimicrobial, antioxidant, and antitumor properties, positions chitosan and its derivatives as promising candidates for applications in pharmaceuticals, food systems, agriculture, and environmental technologies. Continued efforts toward sustainable process development and quantitative performance assessment will be critical to fully realize their potential.

## Data Availability

No new data were created or analyzed in this study. Data sharing is not applicable to this article.
